# The human primary somatosensory cortex encodes imagined movement in the absence of sensory information

**DOI:** 10.1038/s42003-020-01484-1

**Published:** 2020-12-11

**Authors:** Matiar Jafari, Tyson Aflalo, Srinivas Chivukula, Spencer Sterling Kellis, Michelle Armenta Salas, Sumner Lee Norman, Kelsie Pejsa, Charles Yu Liu, Richard Alan Andersen

**Affiliations:** 1 Division of Biology and Biological Engineering, California Institute of Technology, Pasadena, CA USA; 2 Tianqiao and Chrissy Chen Brain-Machine Interface Center, Chen Institute for Neuroscience, California Institute of Technology, Pasadena, CA USA; 3 UCLA-Caltech Medical Scientist Training Program, Los Angeles, CA USA; 4 Department of Neurological Surgery, Los Angeles Medical Center, University of California, Los Angeles, Los Angeles, CA USA; 5 USC Neurorestoration Center, Keck School of Medicine of USC, Los Angeles, CA USA; 6 Department of Neurological Surgery, Keck School of Medicine of USC, Los Angeles, CA USA; 7 Second Sight Medical Products, Sylmar, CA USA; 8 Rancho Los Amigos National Rehabilitation Center, Downey, CA USA

**Keywords:** Cognitive neuroscience, Cortex, Brain-machine interface

## Abstract

Classical systems neuroscience positions primary sensory areas as early feed-forward processing stations for refining incoming sensory information. This view may oversimplify their role given extensive bi-directional connectivity with multimodal cortical and subcortical regions. Here we show that single units in human primary somatosensory cortex encode imagined reaches in a cognitive motor task, but not other sensory–motor variables such as movement plans or imagined arm position. A population reference-frame analysis demonstrates coding relative to the cued starting hand location suggesting that imagined reaching movements are encoded relative to imagined limb position. These results imply a potential role for primary somatosensory cortex in cognitive imagery, engagement during motor production in the absence of sensation or expected sensation, and suggest that somatosensory cortex can provide control signals for future neural prosthetic systems.

## Abstract (web summary)

Matiar Jafari, Tyson Aflalo et al. show that the human primary somatosensory cortex is activated when subjects imagine reaches in a cognitive motor task, but not when they plan movement or imagine a static limb position. These results highlight a role for this region in cognitive imagery and motor control in the absence of sensory information.

## Introduction

Somatosensory cortex (S1) is largely studied and understood in its role as the primary sensory region for processing somatic sensory signals from the body^1,2^. However, recent work highlights a more direct role in motor production: S1 neurons can respond to passive movements alone, active movements alone, or both^3,4^ and neurons become activated prior to movement initiation^3–5^. S1 neurons project to the spinal cord^6,7^, and electrical or optical stimulation of S1 elicits motor movements^2,8,9^. These results suggest a direct role of S1 in the production of motor behavior. However, in many of these studies, it is hard to dissociate whether neural signals reflect motor variables or aspects of sensory processing.

To understand if S1 processes reach intentions in the complete absence of sensation or expected sensation, we recorded neural activity from S1 while a tetrapalegic human participant imagined reaching. Recordings were made from multi-channel microelectrode arrays implanted in S1 as part of an ongoing clinical trial that showed that microstimulation delivered through these same multi-channel arrays evokes localized and naturalistic cutaneous and proprioceptive sensations^10^. The imagined reaching task systematically manipulated fixation, imagined initial hand, and reach target locations at distinct points in the trial^11,12^. We found that S1 neurons encoded movement direction during motor imagery, but did not encode motor plans or imagined arm position. These results establish engagement of S1 during cognitive-motor behavior in the absence of sensations or expected sensations.

## Results

**Fig. 1:**
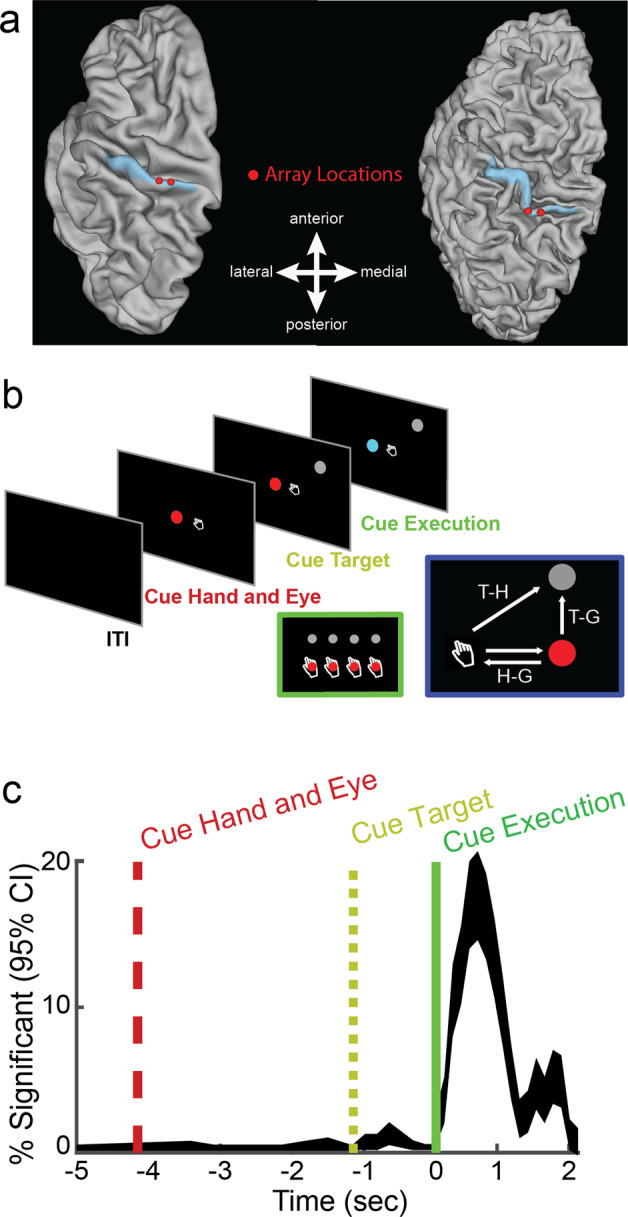
Behavioral task, electrode array location, and percent of the neural population recruited during task epochs. **a** Group-average brain map (left) and brain of subject FG (right) showing location of implanted microelectrode array (red circle) and Brodmann Area 1 (blue shading) in the left hemisphere. **b** Task progression of delayed reference frame reaching task testing all unique combinations of four gaze, hand, and target positions (green inset). Geometry of the reference frame task (blue inset). **c** Percent of task selective units (mean ± SEM *p* < 0.05, FDR corrected, *n* = 652 recorded units). The firing rate of each unit was modeled as a linear function of eye, hand, and target locations and their respective interactions using a sliding window analysis. Units were considered selective if the p-value of the linear fit was significant after false-discovery rate correction.

We recorded 652 channels of single and multiunit activity from multi-channel electrode arrays implanted in S1 of the left hemisphere (Fig. 1a) of a 34-year-old tetraplegic male (FG) during a delayed imagined reaching task. Our paradigm (Fig. 1b), adapted from previous non-human primate (NHP) studies^11,12^, systematically manipulated fixation, imagined initial hand, and reach target locations. Importantly, the participant is capable of moving his eyes and thus can direct his gaze to the fixation targets. However, the paralyzed participant did not move his arm, but instead used motor imagery to imagine moving his right (contralateral) hand to the initial hand cue location and subsequently imagined moving it to the final target. This design allowed us to, one, understand how activity in S1 relates to storing information about arm location, movement plans, and movement execution, and two, characterize the reference frame of these signals, i.e. whether movement variables are coded relative to the initial imagined position of the hand, relative to the eyes, or relative to the body or world.

### Single neurons in human primary sensory cortex are engaged by motor imagery

We performed a sliding window analysis to determine whether and when neurons in S1 become active for our cognitive-motor task. For each unit, we used a linear model with interactions to explain firing rate as a function of fixation, initial imagined hand, and target locations (Fig. 1c, *p* < 0.05 FDR corrected for number of units per time slice, window size: 500 ms, step size: 100 ms). We found negligible selectivity following cueing of the hand and eye positions indicating no neural coding for true eye position or the imagined position of the arm. We also found negligible selectivity following target presentation, indicating no encoding of the spatial location of the target or planning activity related to the upcoming imagined motor action. Finally, we found that a significant proportion of the population was selective following the instruction to initiate the imagined reach. Thus, the sensory cortex is engaged during a cognitive-motor task despite the absence of overt movement and sensory feedback, but only during imagined movement of the limb.

### Imagined movements are coded relative to the initial hand position cue

**Fig. 2:**
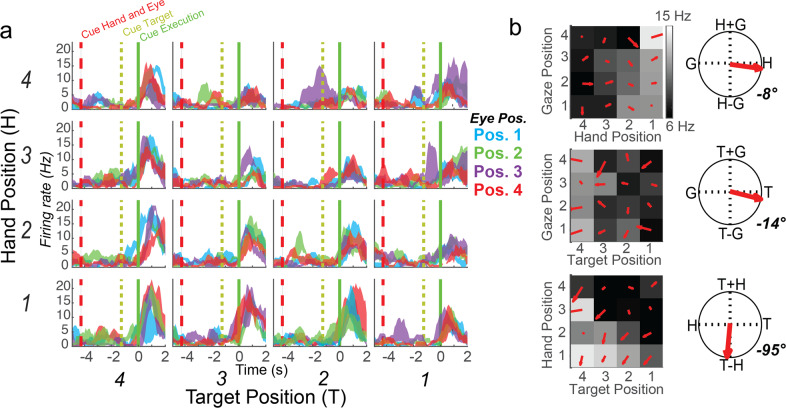
Example S1 Unit illustrating selective responses and response matrices. **a** Peristimulus time histograms for all 64 conditions (3 trials; mean ± SEM). Each of the 16 subplots shows the response of the unit to a particular combination of eye, hand, and target position. **b** Response matrices, gradient field, and gradient resultant orientations for the cell shown in panel **a** during the execution epoch.

**Fig. 3:**
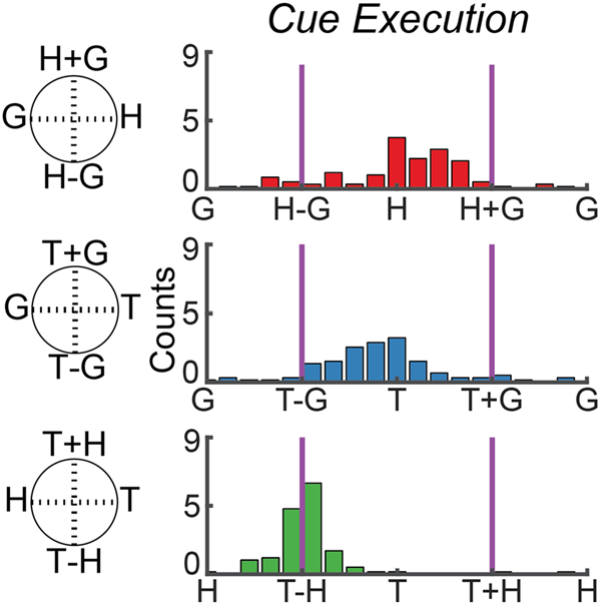
Population summary of single unit gradient analysis. Histograms show gradient resultant orientations for the population of tuned units.

We found that nearly all the neurons selective during the movement execution phase coded movement as the reach vector: the direction of imagined movement of the hand. In other words, selective units coded the location of the target relative to the hand position cue used to instruct the initial imagined hand position (or, by symmetry, hand position cue relative to the target). This result was found using a gradient analysis pioneered in NHPs^11,12^; neural responses for each unit were organized into response matrices where the firing rate is coded for each hand, eye, and target position. A gradient field is then computed which describes how the firing rate is dependent on changes in the three behavioral variables. This dependency is defined along three axes: these axes define how firing rate changes when (1) gaze position changes relative to hand position (and vice versa), (2) gaze position changes relative to target position (and vice versa), and (3) hand position changes relative to target position (and vice versa). Finally, the dependencies are summarized as the resultant, or vector sum, of the gradient field for each of the variable combinations. Critically, the reference frame can only be characterized by the pattern of resultants computed for all three combinations of variables. For example, the triplet of values can be used to determine whether neural activity encodes target position relative to gaze position (T-G), the target position relative to the hand (T-H), the hand position relative to gaze direction (H-G), or some combination of these vectors (Supplementary Fig. 1). A representative neuron coding the position of the target relative to the hand cue and its associated response matrix is shown in Fig. 2a, b. The response matrix is computed from the window of activity following the go cue. Note that looking at the resultant of any one variable pair can be misleading. For instance, in Fig. 2b, the resultant for the gaze and hand combinations shows coding of hand independent of gaze and might be taken to imply neural encoding of hand position. However, the resultant for the hand and target combination shows that any coding for hand position is actually expressed as an encoding of the relative location of the hand and target. Taken together, the unit encodes the relative location of the hand and target, essentially independent of eye position; all three resultants must be considered together. Figure 3 shows the population distribution of response gradient angles computed during the execution epoch for all units with a selective response, based on a linear tuning analysis. The results show that selective units in the recorded neural population strongly encode the reach vector given that essentially all units encode hand relative to target.

**Fig. 4:**
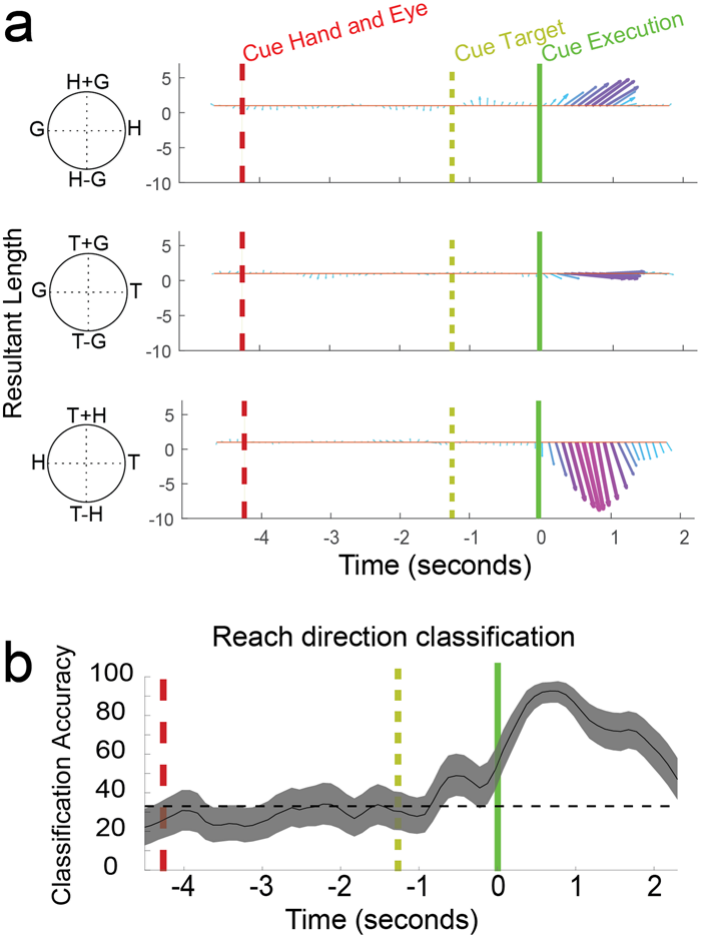
Population dynamics of movement variable encoding. **a** Temporal evolution of reference frame encoding across the population of S1 units. Only the first component (shown) was significant (*p* < 0.05; parallel analysis). Arrow length, width, and color shows tuning strength. Schematic illustration of population gradient analysis is shown in Supplementary Figure 2. **b** Offline analysis depicting cross-validated classification of reach direction initiated from the middle two hand positions to targets located above, to the right, and to the left of the starting hand position (see Supplementary Fig. 3 and Supplementary Fig. 4 for classification details). Sliding window classification performed on a 500-ms window stepped at 100 ms and is shown with mean and 95% bootstrapped confidence interval. Dashed horizontal black line shows chance accuracy (33%).

To verify that this interpretation is an adequate summary of S1 encoding, we used complex principal component analysis (cPCA) to characterize the temporal dynamics of the reference frame of the population as a whole (Supplementary Fig. 2). The gradient analysis described above summarizes the sensitivity of a neuron to behavioral variables using the resultant of the gradient, a 2D vector that can be described by a length and angle. We used cPCA for its capability to handle vector data samples, i.e. described by both a length and angle for each observation^13^. We found that coding of the reach vector strengthens and peaks around 750 ms after the cue to execute the imagined reach (Fig. 4a). Further, only the first cPCA component was significant based on a parallel analysis, a procedure that determines the significance of components by comparing eigenvalues from the recorded dataset versus a random control dataset of the same dimensionality (e.g. *p* variables and n samples) (parallel analysis^14,15^, α < 0.05). This population analysis supports the finding that neural activity correlated with imagined reaching in S1 is dominated by a single homogeneous representation of the reach vector.

### Neural population activity enables decoding of imagined movement direction

Neural encoding of the reach vector can be used to decode the subject’s motor intent relative to the starting location of the effector (Fig. 4b). Ensuring that the neural decoding is designed to interpret neural activity in the correct reference frame is essential to accurate estimation (Supplementary Fig. 3). The geometry of initial hand positions and target locations for our task was selected to determine the reference frame of reach encoding. Importantly, this means that the same target is associated with multiple reach vectors. For this reason, decoding target locations without accounting for initial hand location results in poor classification performance (Supplementary Fig. 3a). To ensure that classification analysis was optimized for decoding the reach vector (i.e. in a hand centered frame), we sub-selected trials with equivalent reach directions and angular separation for classification analysis (Supplementary Fig. 4b). We found that S1 encodes movements accurately with ~92% accuracy for targets separated by 26° and 97% accuracy for reach vectors separated by 44° (Supplementary Fig. 3 and Supplementary Fig. 4).

## Discussion

### Contributions of S1 to motor execution

While the current study demonstrates that S1 is engaged during cognitive imagery, the role of S1 during motor execution remains an open question. It is believed that regions homologous to S1 were the first to bring motor behavior under cortical control^16,17^. In mice, S1 plays a direct role in controlling certain classes of behavior such as whisker retraction^9^. NHP S1 contains monosynaptic descending projections to motor output regions of the spinal cord and thus is able to directly influence motor behavior^7^. These results are consistent with the possibility that the reported S1 responses may play a relatively direct role in motor production, presumably complimentary to other descending motor systems such as primary motor cortex. S1 also has reciprocal connections with the motor cortex, and thus S1 activity may reflect an efference copy of execution signals originating from motor cortices^18^. This could mediate coordination for descending control and/or may play a role in processing afferent sensory inflow. Lesions to human or NHP S1, while preserving the basic ability to move, nonetheless result in profound motor deficits^19^. Whether these deficits are exclusively related to difficulties in planning and executing movements in the absence of sensory input or also reflects a direct loss of cortex responsible for motor production remains unclear^20^.

### Interpreting the percent of selective units

This study establishes that S1 encodes the direction of imagined reaches relative to the starting location of the effector. However, the spatial layout of the task, while well designed for the study of spatial reference frames^11,12^, involves a highly restricted angular range of movement directions (e.g. up, up and to the right, up and to the left). As a consequence, our study likely underestimates the percentage of the S1 neural population selective for imagined movements. For example, neurons with preferred directions of down and to the right, or in and out of the movement plane, will likely not show up as significant in our study. Further, recent single-unit studies in humans have shown that localized populations of neurons are selective for multiple segments of the body and these different segments engage distinct populations of neurons^21–23^. Future studies, testing more effectors and a fuller range of movements, are necessary to better estimate absolute percentages of S1 populations engaged by motor imagery.

### Do neural signals reflect the correlates of motor imagery

Interpreting neural representations during imagery presents challenges due to the inability to directly determine what the subject is imagining. For example, in our study we ask the subject first to imagine his hand at the hand position cue and then to imagine moving his hand to the instructed target. However, we are unable to independently verify that the participant is performing the task as instructed. This opens the possibility that neural signals in S1 may not be coding imagined arm movements per se, but instead generalized effectors movements, cognitive spatial variables, or other correlates of motor behavior. However, our previous results studying imagery at the level of single neurons in humans show effector movement imagery recruits responses in the same cells that also respond to the actual movement of the effector and that imagery of different effectors engages distinct populations of neurons^21,22^. These results argue that neural responses can be interpreted as the correlates of imagery involving specific effector movements. These results are not specific to spinal cord injured subjects as work in motor intact individuals demonstrate a shared neural substrate between overt and imagined actions^24^. Further, the highly specific timing of neural encoding, strong parametric modulation by intention, and high trial-to-trial consistency of responses (as evidenced by high classification accuracy) support consistent trial to trial compliance by the participant. Taken together, S1 signals likely reflect the correlate of imagined movement of the arm with respect to its starting location.

### Motor imagery in primary somatosensory cortex

To our knowledge, our study is the first to probe single neuron responses to motor imagery in the primary somatosensory cortex of a human subject. Previous work has studied imagery using functional neuroimaging and generally shows either a strong reduction of activity when comparing imagined to executed actions^25–27^ or no significant activation^28,29^. A reduction in neural responses to imagery should be expected given the absence of peripheral sensory input, the primary driving inputs to the region. From this perspective, it is somewhat surprising that single units demonstrate the degree of temporal and spatial precision demonstrated in this study. A possible concern is that our results are unique to individuals who have lost their main peripheral input due to spinal cord injury. However, recent findings from our lab and others have shown these representations are largely stable and reorganization does not result in the production of novel functional responses^10,30–32^. Further, our results are consistent with studies in motor intact preparations demonstrating movement-related activity^3–5,9^. Finally, as discussed above, fMRI frequently reveals imagery related activity in S1. Thus, we do not think our results are primarily a function of loss of peripheral input, although it is possible that spinal cord injury may enhance responses in primary sensory cortices^33,34^.

### S1 as a potential source of neural prosthetic signals

We placed electrode arrays in S1 to provide somatic sensations using electrical stimulation. Intriguingly, directional selectivity and the ability to accurately classify movement direction suggests that S1 can also be used to control a neural prosthetic or augment control signals from additional brain regions^35–37^. However, important questions about the viability and performance of S1 signals for closed-loop neural control remain. For example, the current study does not demonstrate that S1 encodes dynamic properties of movements such as speed or that S1 can simultaneously encode multiple control signals that would enable more complex behaviors such as control of cursor movements and clicking. The properties of S1 signals during closed-loop control remains an important and unexplored question that will require future study.

## Conclusion

Our study challenges the classical understanding of S1 as an early cortical processing station for incoming sensory information by providing evidence for a possible role in motor production and cognition. S1 neurons tracked motor execution intentions in the complete absence of sensation exclusively during imagined execution. We found negligible activity while the subject maintained the position of the limb in memory, fixated distinct targets, or planned movements. S1 activity coded intended movements relative to the imagined initial state of the effector. This activity accurately predicted movement direction and thus may provide neural signals that can assist in the closed-loop neural control of prosthetic effectors.

## Methods

### Participant Information

Neural recordings were made from participant FG, a tetraplegic 32-year-old male with a complete C5/C6 spinal cord injury. FG was implanted 1.5 years post-injury for a clinical trial of a BMI system consisting of intracortical stimulation and recording. Neural recordings for the current study were acquired 1-year post-implantation. All of subject FG’s sensations and motor ability are consistent with the level of the sustained injury. The subject remains intact for all other motor control and sensations above the level of injury. Surgical implantation took place at Keck Hospital of USC.

Experiments were conducted in the framework of an ongoing neural prosthetics clinical study (ClinicalTrials.gov identifier: NCT01964261) and were in compliance with all relevant clinical regulations. We obtained informed consent after explaining the objectives of the study and the possible risks involved. The study and all procedures were approved by the Institutional Review Boards (IRB) of the California Institute of Technology (Caltech), the University of Southern California (USC), and Rancho Los Amigos National Rehabilitation Hospital (RLA).

### Surgical planning and implantation

In brief, functional magnetic resonance imaging (fMRI) was used to measure the BOLD response while FG performed imagined reaching and grasping movements in response to visual cues^10,22^. The statistical parametric analysis guided the selection of implant locations in the left hemisphere of the ventral portion of the premotor cortex (PMv), the supramarginal gyrus (SMG), and the somatosensory cortex (S1). PMv and SMG were implanted with 96-channel Neuroport microelectrode arrays (Blackrock Microsystems, Salt Lake City, UT). S1 was implanted with two 7×7 microelectrode arrays (48 channels per array, Blackrock Microsystems, Salt Lake City, UT) on the post-central gyrus. Figure 1b shows the implantation locations for the two arrays. In addition, we estimated the anatomical location of the implantation of S1 in terms of Brodmann’s Area. To this end, we used Freesurfer^38^ to perform a surface reconstruction of the individual subject’s anatomy. The subject’s anatomy was then registered to the 164K fs-lr group-average template using Connectome Workbench^39^. The subject’s implants were determined to be localized to Brodmann’s Area 1 (BA1) according to the composite template of Van Essen et al 2012^39^ as visualized within Connectome Workbench. Localizing the areal boundaries of BA1 within the individual subject requires the registration of the individual subject’s surface anatomy to a group-average atlas. We therefore show implant locations both as they appear on the individual subject’s brain surface as well as where the arrays are estimated to be located on the fs-lr group-average template brain (Fig. 1a).

### Reference frame task

Experimental sessions with subject FG were performed at Rancho Los Amigos National Rehabilitation Center (RLA). FG performed the task in a dimly lit room seated in his motorized wheelchair. Task stimuli were viewed on a 47-inch LCD monitor with the screen occupying approximately 45° of visual angle. The subject was asked to minimize head movements throughout the task. At the beginning of each trial, FG was presented with a fixation cue and a hand position cue. Each cue could be positioned at one of four locations resulting in 16 possible hand and eye configurations. FG was able to move his eyes and thus fixate the fixation cue as verified using eye tracking. In contrast, FG did not position his actual hand at the location of the hand cue, but instead FG imagined moving his right (contralateral) hand to the cued location and maintained imagery of his hand until he was cued to make a reach (Fig. 1b, “go” cue). After 3 s a reach target cue was shown at one of four spatial locations arranged parallel to and above the cued eye and hand positions. The target cue was shown for 1.25 s during which the subject continued to hold their gaze and imagined hand positions. A change in the color of the fixation marker instructed the subject to begin an imagined reach to the cued target location. The subject was asked to make an imagined reach and maintain the imagined ending position (target location) until the execution epoch was over (2 s). The execution epoch was then followed by an inter-trial interval (ITI) of 2 s. A schematic representation of the task is shown in Fig. 1b.

Experimental data were collected in three experimental runs. Each run consisted of a total of 64 trials, one trial for each unique combination of the four eye, hand, and target positions. This resulted in 192 total trials, 3 repetitions for each unique trial type. Each experimental session was separated out by at least a week.

### Neural Recordings

Neural activity from each array was amplified, digitized, and recorded at 30 kHz using the Neuroport neural signal processor (NSP). The Neuroport system, comprised of the arrays and NSP, has received FDA clearance for less than 30 days of acute recordings. However, for purposes of this study we received FDA IDE clearance for extending the duration of the implant (IDE number: G130100).

Putative waveforms were detected at thresholds of −3.5 times the root-mean-square after high pass filtering the full bandwidth signal (sampled at 30 kHz), using the Blackrock Central software suite (Blackrock Microsystems). Waveforms consisted of 48 samples, 10 prior to threshold crossing and 38 samples after. These recordings were then sorted (both single and multiunit) using k-mediods clustering using the gap criteria to estimate the total number of clusters^21,40^. Offline sorting was then reviewed and adjusted as needed following standard practice^41^. On average across 4 days of recordings in S1 we analyzed 163 sorted units per session. All sorting was done prior to analysis and blind to channel or unit responses found during the task. Further spike sorting methods can be found in Zhang and Aflalo et al., 2017.

### Eye tracking

Subject FG’s eye position was monitored using a 120 Hz binocular eye-tracking system (Pupil Labs, Berlin, Germany). If the subject’s gaze shifted off the cued eye position the task was terminated and restarted to ensure that gaze position was correct and remained fixed to the cued eye position throughout each appropriate epoch for a run (64 consecutive trials). Eye positions were synced to the task and allowed online determination of eye position. We instructed the subject to maintain a constant head position and to only move his eyes to fixate the target. However, head position was not monitored and more conservatively we can say that we manipulated gaze as opposed to eye position proper. In either case, our results showed no dependences on eye/gaze and thus the distinction is not especially important given the pattern of results.

### Linear analysis for tuning (Fig. 1c)

We defined a unit as selective if the unit displayed significant differential modulation for our task variables as determined by a linear regression analysis: We created a matrix that consisted of four indicator variables for each unique behavioral variable (e.g. one indicator variable for each of the four initial hand positions) resulting in 12 indicator variables. Firing rate was estimated as a linear combination of these indicator variables and their interactions: FR is firing rate, Xc is the vector indicator variable for condition c, ß_c_ is the estimated scalar weighting coefficient for condition c, and ß_0_ is the intercept.

$${\rm{FR}} = \mathop {\sum}\limits_c {\beta _c} X_c + \beta _0.$$

Linear analysis was performed over a sliding window throughout the interval of the task. Windows were 750 ms in duration and window start times were stepped every 500 ms. Significance of each fit was determined using the *p*-value of the F-test of overall significance for the linear analysis (*p* < 0.05, FDR corrected for number of units). Units that were found to be significant in this analysis were then determined to be selective and further analyzed in the reference frame analysis.

### Reference frame analysis: gradient analysis (Fig. 2)

Gradient analysis was used to quantify how changes in the behavioral variables changed the firing rate of each unit when comparing across each unique combination of variable pairs (Hand-Gaze (HG), Target-Gaze (TG), and Target-Hand (TH))^11,12^: For each tuned unit (based on the p-value of the linear regression model described above) we created a four by four matrix (response matrix) representing neural activity for each unique combination of two behavioral variables; thus, for example, the value at the HG response matrix location [*x*,*y*] would be the neural activity recorded for hand position x and gaze position y averaged across trial repetitions and repetitions acquired for the different target positions. Gradients were determined using the gradient function in Matlab 2019a (Mathworks Inc, Natick, MA). For each gradient, a resultant angle and length was computed to summarize the net direction and magnitude of change across the entire response matrix (Supplementary Fig. 1). However, gradients often show a symmetrical pattern that would result in cancellation of symmetrical angles (Supplementary Fig. 1a). To avoid this, we double each angle in the matrix and represent each angle from 0° to ±180°. Therefore, the summed resultant angle is represented by 0° for gradients oriented left and right, ±180° for gradients oriented up and down, and −90° for gradients oriented along the diagonal (Supplementary Fig. 1a). The summed resultant angle and length however cannot be mapped directly onto the response matrix; thus, we have notated the appropriate variable and combinations of variables to help with interpretation. For example, in Supplementary Figure 1a hand only (H) modulation would be found at ±180°, gaze only (G) modulation is seen at 0°, H+G at 90°, and H-G at −90°. Therefore, we can use the angle of the resultant angle as a proxy for overall orientation bias for a variable or variable pair.

### Population reference frame analysis (Fig. 3)

We used population-level dimensionality reduction analyses to determine the most common modes of reference frame encoding over time. This was done in a three-stage process (see Supplementary Fig. 2): (1) Initial principal component analysis (PCA) on the time-varying activity of the neural population, (2) reference frame analysis on each time point of the resulting principal components, (3) complex principal component analysis (cPCA) on the resultant angles and magnitudes. The initial PCA was used to denoise and improve the calculation of reference frames at the level of the population. In order to perform PCA analysis we constructed a matrix of neural data *D* that was (*n*) by (*t* * *c*) in size, with *n* being the number of neurons, *t* being the number of time points, and *c* being the number of conditions. For each neuron, activity was averaged across repetitions of the same condition within a 100 ms window. The reference frame analysis was then applied to each temporal window for the first 20 principal components. Note that following the initial PCA, the population activity still carries detailed information about neural selectivity properties unrelated to the reference frame proper (e.g. preferred directions of movement or preferred hand locations). Thus multiple principal components may have the same reference frame, but simply prefer a different movement direction. Computing the reference frame at this stage extracts the population level reference frame, abstracting away tuning preference differences. The final cPCA was then used to capture the main reference frame modes once the detailed aspects of tuning (e.g. such as preferred direction of response) were abstracted away by the reference frame analysis. We used cPCA given the fact that dimensionality reduction was performed on the resultant vectors, values with both a magnitude and angle. We converted all resultant angles and lengths into complex numbers to apply cPCA^13^. We used parallel analysis to determine which components from this dimensionality reduction were significant.

### Discrete classification (Fig. 3b, Suppementary Figs. 3,  4)

Offline classification was performed using linear discriminate analysis. The classifier took as input a vector comprised of the number of spikes occurring within a specified time epoch for each sorted unit. The following assumptions were made for the classification model: (1) the prior probability across the classes was uniform, (2) the conditional probability distribution of each feature on any given class was normal, (3) only the mean firing rates differ for each class (the covariance of the normal distributions were the same for each class), and, (4) the firing rates of each input are independent (covariance of the normal distribution was diagonal). Reported performance accuracy was based on leave-one out cross-validation. To compute the temporal dynamics of classification accuracy, the neural data were first aligned to a behavioral epoch (e.g. cue execution onset). Spike counts were then computed in 500 ms windows spaced at 100 ms intervals. Classification accuracy was computed independently for each time bin and bootstrapped resampling was used to compute 95% confidence bounds. In the supplementary materials (S3, S4), we performed a number of classification analyses on a single time window. This window used average neural activity from 0.25 seconds to 1.25 s after the go cue to capture the neural response during the period of motor imagery.

### Neuron-dropping curve analysis (Supp Fig. 4)

Neuron-dropping curves were constructed to understand the strength of neural coding for movement direction as a function of neural yield. To construct the random neuron-dropping curves of Supplementary Figure 4, we computed cross-validated decode accuracy using LDA classification (described above) for test populations of neurons. Each test population was generated by randomly sub-selecting, without replacement, the specified number of units from the entire ensemble of recorded units. For each population size, units were randomly drawn and cross-validated accuracy was computed 100 times to allow estimation of the variability in accuracy. Results are shown as the individual accuracies, mean, and bootstrapped confidence interval.

### Statistics and reproducibility

Multiple regression analysis for determining if a unit was significantly fit was done using a linear model (*fitlm* in MATLAB). Coefficients of the linear model were then determined to be significant using an *F*-test (*coefTest* in MATLAB). Significance was then corrected for using the false-discovery rate (*p* < *0.05)*.

Gradients were determined using the *gradient* function in MATLAB. For each gradient, a resultant angle and length were computed to summarize the net direction and magnitude of change across the response matrix. Due to symmetrical patterns that would result in cancellation of symmetrical angles we double each angle in the matrix and represent each angle from 0° to ±180°.

Dimensionality reduction analysis was done using the *PCA* function in MATLAB. For subsequent complex PCA analysis each respective resultant angle and magnitude was then converted into a complex number: c is the desired complex number, *r* is the magnitude of the resultant angle, *i* is an imaginary number equal to $\sqrt { - 1}$, and θ is the resultant angle in radians. $c = r \times e^{(i \times \theta )}$

Parallel analysis is a Monte-Carlo based simulation that compares observed eigenvalues with those obtained from uncorrelated variables^14,15^. Components were retained if the associated eigenvalue was larger than the 95th percentile of the distribution of eigenvalues, derived from the randomization of data (10,000 shuffles).

## Supplementary information

Supplementary Information

Description of Additional Supplementary Files

Supplementary Data 1

## Data Availability

All primary behavioral and neurophysiological data are archived in the Division of Biology and Biological Engineering at the California Institute of Technology and are available from the corresponding author on reasonable request. Source data underlying plots shown in figures are provided in Supplementary Data 1.

## Code availability

All custom-written analysis code is available from the corresponding author on reasonable request.
