# Supplementary material for: The human primary somatosensory cortex encodes imagined movement in the absence of sensory information
Source: Commun Biol. 2020 Dec 11;3:757. doi: 10.1038/s42003-020-01484-1 (PMC7732821; doi:10.1038/s42003-020-01484-1)
Supplement: Supplementary file 1 — Supplementary Information [file 42003_2020_1484_MOESM1_ESM.pdf]

Supplementary Materials for

The human primary somatosensory cortex encodes imagined movement in the absence of sensory information

Matiar Jafari [†,1,2,3], Tyson Aflalo[†,1,2,‡], Srinivas Chivukula[1,4], Spencer S Kellis[1,2,5,6], Michelle Armenta Salas[7], Sumner L Norman[1,2], Kelsie Pejsa[1,2], Charles Y Liu[,5,6,8], Richard A Andersen[1,2]

‡ Correspondence to taflalo@caltech.edu.

† Contributed Equally

Affiliations:

1) Department of Biology and Biological Engineering, California Institute of Technology, Pasadena, United States
2) Tianqiao and Chrissy Chen Brain-Machine Interface Center, Chen Institute for Neuroscience, California Institute of Technology, Pasadena, United States
3) UCLA-Caltech Medical Scientist Training Program, Los Angeles, United States
4) Department of Neurological Surgery, Los Angeles Medical Center, University of California, Los Angeles, Los Angeles, CA, United States
5) USC Neurorestoration Center, Keck School of Medicine of USC, Los Angeles, United States
6) Department of Neurological Surgery, Keck School of Medicine of USC, Los Angeles, United States
7) Second Sight Medical Prod. Sylmar, CA, United States
8) Rancho Los Amigos National Rehabilitation Center, Downey, United States

This PDF file includes:

Supplementary figure 1: Idealized Unit Responses Displaying Eye-Hand-Target Tuning for Three Different Reference Frames.

Supplementary figure 2: Graphical Illustration of Complex PCA Processing for establishing population level reference frames.

Supplementary figure 3: Population decoding of spatial targets and reach directions in S1.

Supplementary figure 4: Classification analysis for reaching movements separated by 44 and 26 degrees.

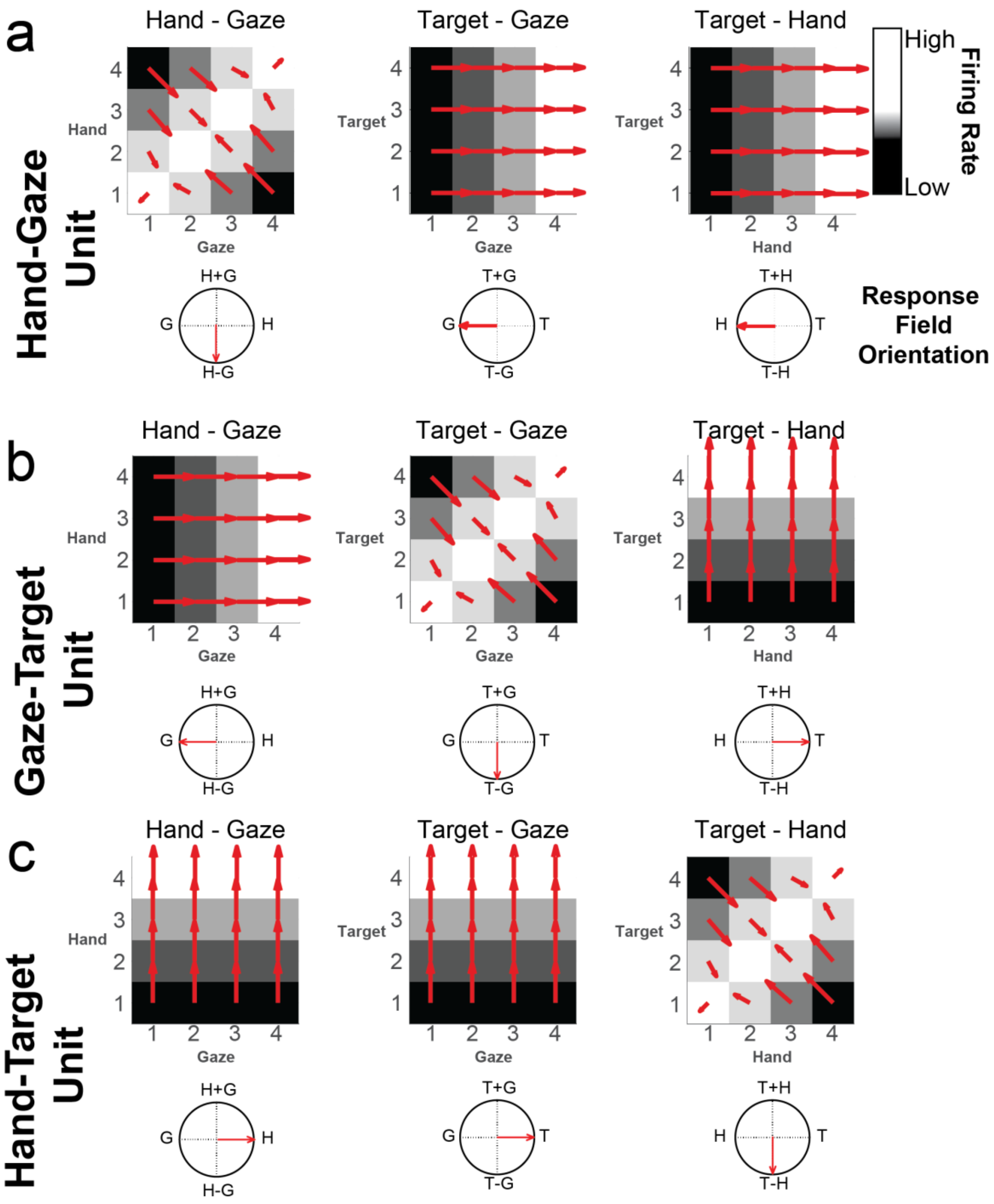

Figure S1. Idealized Unit Responses Displaying Eye-Hand-Target Tuning for Three Different Reference Frames. **a)** Hand-Gaze unit encoding the relative location of the hand and point of gaze. **b)** Gaze-Target unit encoding the relative location of the target and point of gaze. **c)** Hand-Target unit encoding the relative location of the hand and target. The response field orientations for all units are shown for each of the three variable pairs along the horizontal beneath each response matrix. Gradient analysis, and the subsequently derived resultant angle (±180° and 0° point left and right, respectively) and length, quantifies how firing rate responses can be attributed to each behavioral variable (HG, TG, and TH).

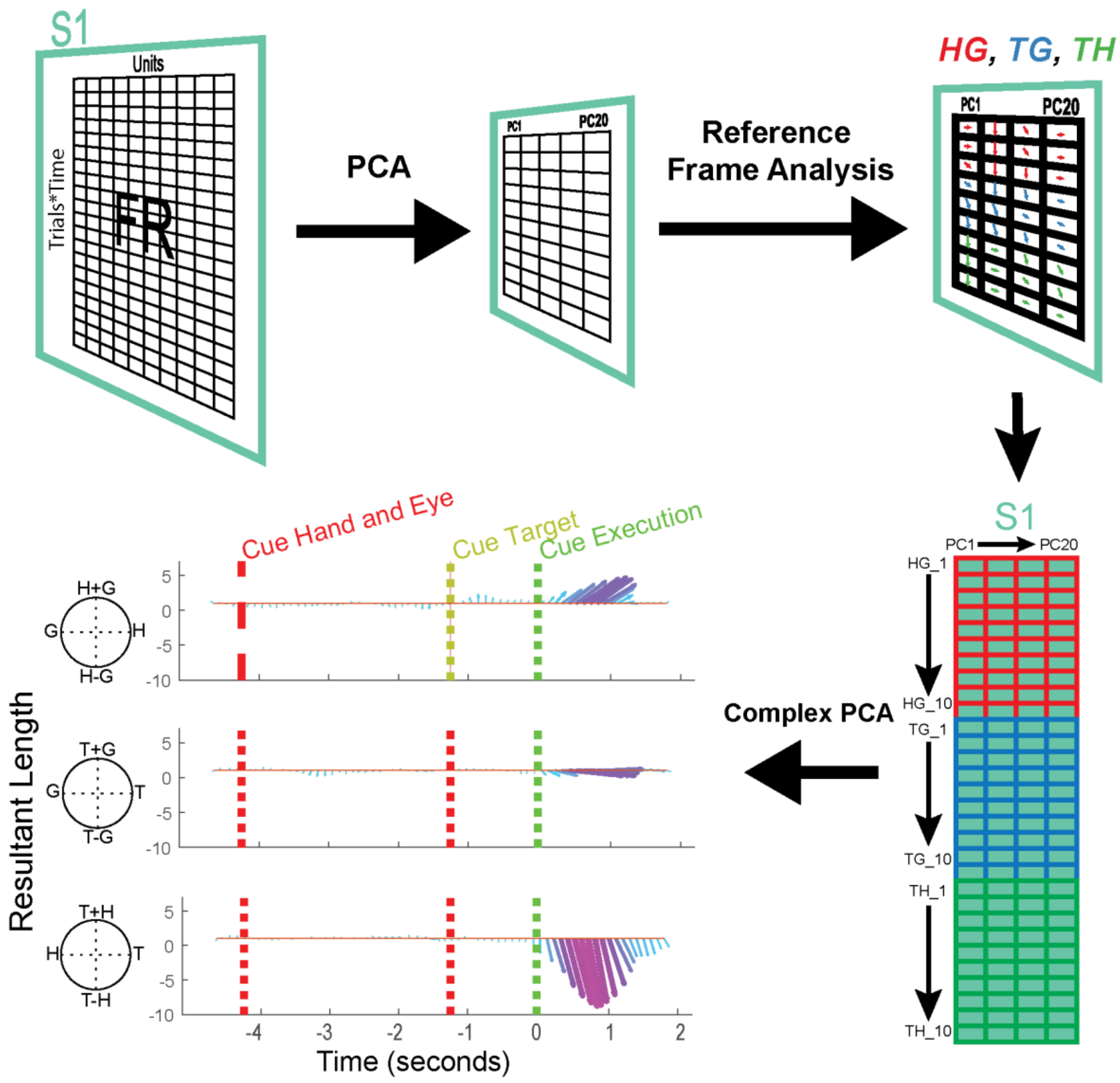

Figure S2. Graphical Illustration of Complex PCA Processing for establishing population level reference frames. Initial principal component analysis (PCA) on the time varying activity of the neural population is used to denoise and improve the calculation of reference frames at the level of the population. This is followed by reference frame analysis on each time point of the resulting principal components. Finally, complex principal component analysis on the resultant angles and magnitudes from the reference frame analysis is used to quantify population reference frames.

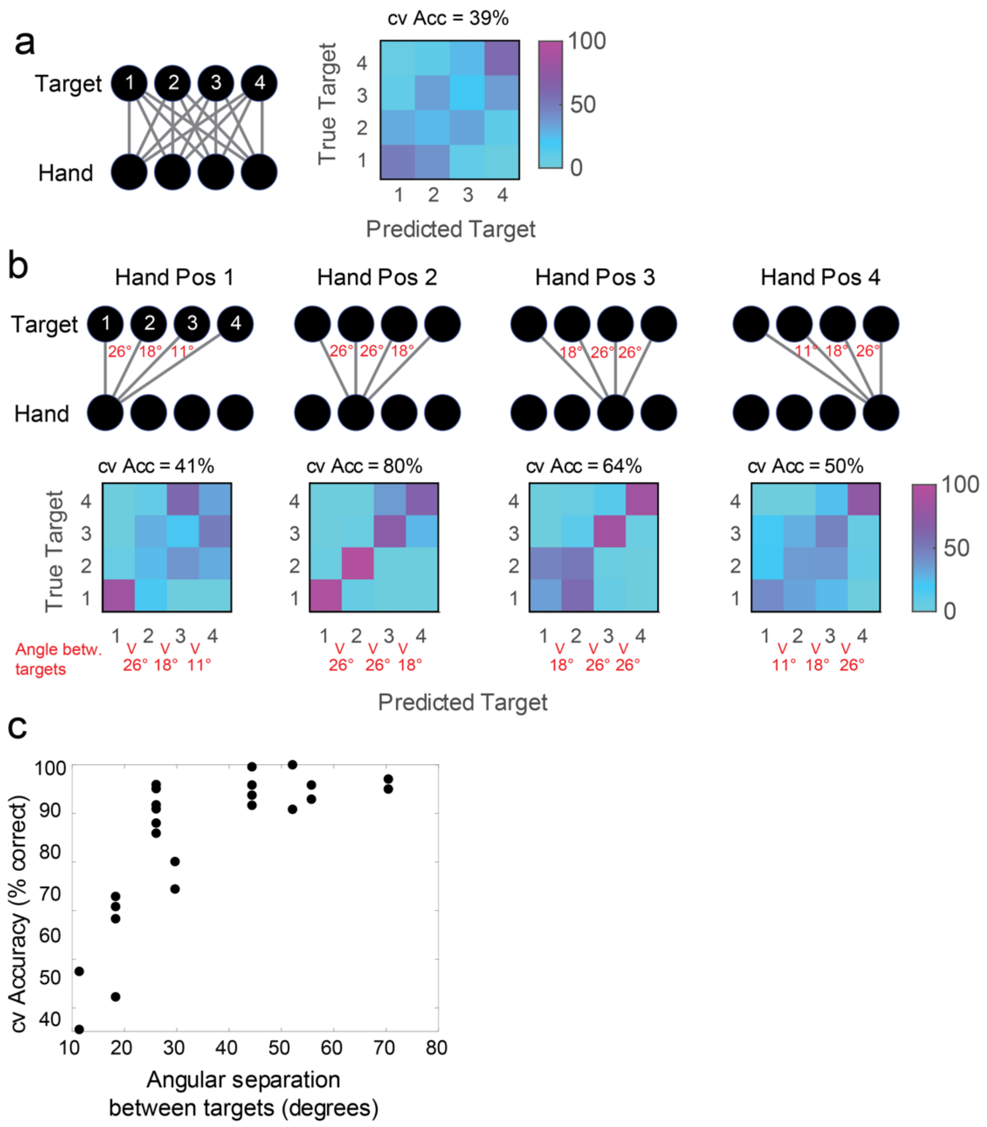

Figure S3. Population decoding of spatial targets and reach directions in S1. All accuracy calculations were performed using leave-one out cross-validation on average activity during the presumed time of active movement imagery, 0.25 to 1.25 seconds after the go cue. **a)** Left: Schematic illustration of starting hand and target positions (black circles) along with movement vectors (grey lines). Right: Confusion matrix for absolute target location (without factoring in initial imagined hand position). Results show low cross-validated accuracy and only extreme target positions with correct inferred labels. Low-classification accuracy is consistent with hand-centered encoding of imagined reaches because the same target is associated with different movement directions depending on the imagined starting hand position. **b)** Top row: Schematic illustration of starting hand and target positions (black circles) along with movement vectors (grey lines) with trials now split by initial hand position. Red text indicates angular separation between two targets given starting hand position. Bottom row: Confusion matrix for target location for each initial hand position. Title shows cross-validated accuracy across all targets for an imagined initial hand position. Splitting trials based on starting hand position ensures that each target is associated with a unique movement direction. Confusion matrices provide evidence that the accuracy in classifying targets varies as a function of angular separation of the reach vectors (vector connecting starting hand position and target location). **c)** Classification accuracy varies as a function of the angular separation between targets. Each point represents the cross-validated accuracy of the pairwise classification between two targets for a fixed hand position. All possible pairwise classifications for two targets from the same starting hand position are shown.

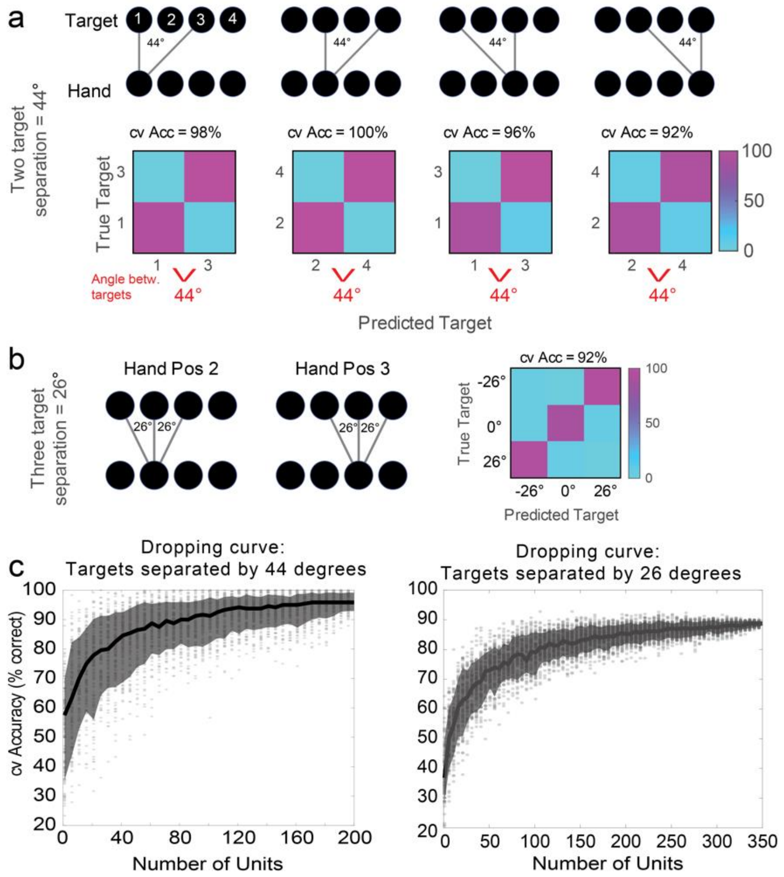

Figure S4. Classification analysis for reaching movements separated by 44 and 26 degrees. **a)** Classification of targets separated by 44 degrees, approximately the same angular separation for eight target center-out tasks. **Top row:** Schematic illustration of imagined starting hand and target positions (black circles) along with imagined movement vectors (grey lines) with trials split based on initial hand position. **Bottom row:** Confusion matrix for target location for each initial hand position. High classification accuracy for targets separated by 44 degrees demonstrates accurate population encoding of reach direction. **b)** Similar to panel a but for three targets separated by 26 degrees. Here, imagined movements are in the same direction, whether starting from imagined hand position 2 or 3, and grouped together for classification analysis. Data from the same set of movement directions was used for time-resolved classification in figure 4b. **c)** Classification neuron dropping curves showing how cross-validated classification accuracy for targets separated by 44 degrees (panel a) and classification for targets separated by 26 degrees (panel b). Dropping curves are constructed by randomly sampling n units (x-axis) and computing cross-validated accuracy. The process is repeated 100 times each with a different realization of the n units. Individual points show results for each sample of n units. Mean and 95% percentile range are shown as the black line and gray interval respectively.
