# Supplementary material for: The human primary somatosensory cortex encodes imagined movement in the absence of sensory information
Source: Commun Biol. 2020 Dec 11;3:757. doi: 10.1038/s42003-020-01484-1 (PMC7732821; doi:10.1038/s42003-020-01484-1)
Supplement: Supplementary file 2 — Description of Additional Supplementary Files [file 42003_2020_1484_MOESM2_ESM.pdf]

Description of Additional Supplementary Files

File Name: Supplementary Data 1
Description: Source data for generating figures
